# Frailty trajectories preceding dementia: an individual-level analysis of four cohort studies in the United States and United Kingdom

**DOI:** 10.21203/rs.3.rs-4314795/v1

**Published:** 2024-04-29

**Authors:** David Ward, Jonny Flint, Thomas Littlejohns, Isabelle Foote, Marco Canevelli, Lindsay Wallace, Emily Gordon, David Llewellyn, Janice Ranson, Ruth Hubbard, Kenneth Rockwood, Erwin Stolz

**Affiliations:** The University of Queensland; The University of Edinburgh; Translational Epidemiology Unit (TEU); University of Colorado Boulder; Sapienza University; University of Cambridge; The University of Queensland; University of Exeter; University of Exeter; The University of Queensland; Nova Scotia Health; Medical University of Graz

## Abstract

Frailty may represent a modifiable risk factor for dementia, but the direction of that association remains uncertain. We investigated frailty trajectories in the years preceding dementia onset using data from 23,672 participants (242,760 person-years of follow-up, 2,906 cases of incident dementia) across four cohort studies in the United States and United Kingdom. Bayesian non-linear models revealed accelerations in frailty trajectories 4–9 years before incident dementia. Among participants whose time between frailty measurement and incident dementia exceeded that prodromal period, frailty remained positively associated with dementia risk (adjusted hazard ratios ranged from 1.20 [95% confidence interval, CI = 1.15–1.26] to 1.43 [95% CI = 1.14–1.81]). This observational evidence suggests that frailty increases dementia risk independently of any reverse causality. These findings indicate that frailty measurements can be used to identify high-risk population groups for preferential enrolment into clinical trials for dementia prevention and treatment. Frailty itself may represent a useful upstream target for behavioural and societal approaches to dementia prevention.

## 1. INTRODUCTION

Research on dementia causes is dominated by Alzheimer’s disease, which focuses on singular disease mechanisms that do not account for symptomology in most cases ^1,2^. However, as dementia most commonly arises in older people with mixed age-related neuropathologies ^1,2^, processes of ageing are implicated in shaping disease susceptibility. This perspective is supported by evidence linking changes in the biological hallmarks of ageing with differences in dementia risk and has given rise to the development of novel anti-ageing approaches to neurodegenerative conditions ^3,4^, for which phase 1 trial results are now being reported ^5^. In addition to informing drug discovery, better understanding the complex relationship between ageing and late-life dementia may be leveraged into behavioural and societal approaches to dementia prevention. For the optimal development of such approaches, a readily measurable target that captures biological age and causally associates with incident dementia is required. Accumulating evidence indicates that frailty may be a viable candidate for that role ^6–8^.

Frailty can be understood as a gradable health state that increases risk for adverse health outcomes independently of chronological age and reflects differences in the accumulation of age-related health deficits ^9^. At any age, a higher degree of frailty is associated with higher all-cause mortality to a greater degree than are common lab-based estimates of biological age ^7,10^. Assessing frailty may therefore provide an accessible means of estimating biological age ^6,9^, with broad relevance to disease prognosis and care planning ^7^. Epidemiological reports using data from independent cohorts have consistently shown that dementia occurs more frequently among those individuals who have a higher degree of frailty ^8,11–14^. These associations persist after adjusting for chronological age and other possible confounding factors, such as sex and educational attainment. Even so, the current evidence base falls short of allowing a causal interpretation of the association of frailty with dementia due to the unresolved possibility of reverse causality. For example, Alzheimer’s disease is thought to have a long preclinical phase (up to 15–20 years) ^15,16^, with subtle changes in health, function and behaviour detectable in the years prior to dementia diagnosis ^16–20^. Therefore, among people assessed as being without dementia at the time of frailty measurement, subclinical changes in health and function may already be reflected as a higher degree of frailty and consequently confound the subsequent detection of a causal relationship between frailty and incident dementia.

In the absence of randomised controlled trials, cohort studies together with statistical approaches using backwards timescales can detail the temporal nature of dementia risk factors with dementia onset ^21,22^. That approach and investigation of its consequences on risk associations have not yet been applied to frailty. Understanding the dynamics of frailty trajectories in the years before dementia can test frailty as an upstream target in efforts to reduce dementia incidence. It may also inform optimal approaches to the targeted recruitment of high-risk populations into clinical trials for dementia prevention and treatment. Using four cohort studies of health, cognition and ageing, we aimed to clarify the relationship between frailty and incident dementia while considering the possibility of reverse causality. To achieve this, we pursued two objectives: (1) determine when an acceleration in the accumulation of frailty due to impending dementia is first observable and (2) measure the association of frailty and dementia risk after controlling for any impact of that pre-dementia frailty acceleration period. The null hypothesis is that any increased risk of dementia in relation to frailty would not hold when frailty measurement occurred before the pre-dementia frailty acceleration period.

## 2. METHODS

### 2.1. Datasets

We analysed participant data from four large cohort studies: the English Longitudinal Study of Ageing (ELSA), Health and Retirement Study (HRS), Rush Memory and Aging Project (MAP), and National Alzheimer’s Coordinating Center (NACC). ELSA is a longitudinal panel study of a representative sample of community-dwelling adults aged 50 years or older in England ^23^. HRS, a longitudinal panel study, surveys a representative sample of older adults in the United States ^24^. MAP is a clinical-pathological cohort study of older adults in Illinois, United States ^25^. NACC collects participant data contributed by Alzheimer’s Disease Research Centers (ADRCs) in the United States using standardised methods ^26^. Details of study methodology and data access are included in Supplementary Information 1.

**Figure 1:**
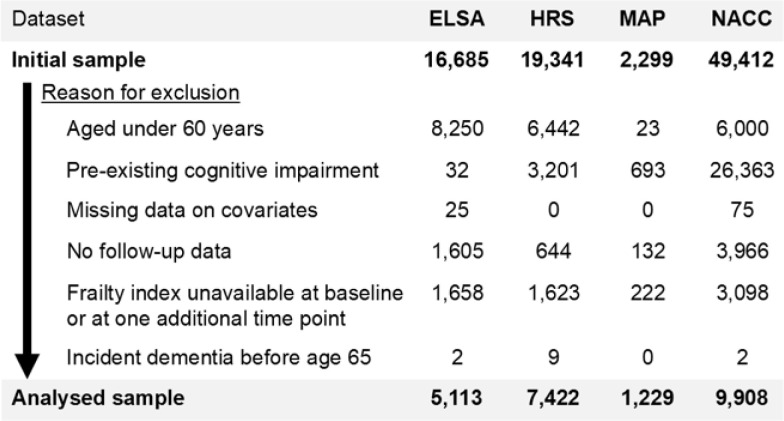
Participant exclusions and analytical samples. ELSA, English Longitudinal Study of Ageing; HRS, Health and Retirement Study; MAP, Rush Memory and Aging Project; NACC, National Alzheimer’s Coordinating Center.

Participants were included if they were aged 60 years or over at baseline, were without cognitive impairment, had data available on age, sex and education level, had some follow-up data, and had sufficient data to calculate a frailty index score at baseline assessment and at least one additional timepoint prior to incident dementia or censoring (Fig. 1). Frailty index scores were only calculated where participants had information available on at least 30 deficits used in that study’s frailty index ^27^. To remove the influence of early-onset dementia cases that often occur exclusively due to genetic causes ^28^, participants were also excluded if they developed dementia before age 65 years.

### 2.2. Incident dementia

Given that mixed dementia is what occurs chiefly in late life ^1,2^, the study outcome was all-cause dementia. The method of determining this outcome differed between studies. In ELSA, classifications were derived through either a self-report of physician diagnosis of dementia or a mean score of ≥ 3.4 on the 16-item Informant Questionnaire on Cognitive Decline in Elderly (IQCODE) completed by family members/caregivers, which represents a decline in the ability of daily function compared to two years prior of a magnitude indicating dementia ^29^. In HRS, classifications of dementia were obtained using the Langa-Weir Classification of Cognitive Function method, which applies validated cut-points to summary scores obtained from a range of cognitive tests (scores ranged from 0–27; scores of 0–6 indicated the presence of dementia) ^30^. In MAP, presumptive diagnoses of dementia and Alzheimer’s disease were calculated via an algorithmic decision tree using accepted clinical criteria and confirmed by a clinician ^31^. In NACC, either a consensus team or a single physician used standard diagnostic criteria to classify participants as having all-cause dementia ^31,32^.

### 2.3. Frailty measurement

Frailty was the main exposure in this study, with each participant’s degree of frailty quantified using retrospectively calculated frailty index scores. The frailty index approach was used due to its value in predicting adverse health outcomes relative to other common approaches to frailty assessment ^33^. The frailty index is a measure of health state, combining information from multiple physiological systems and closely reflecting an individual’s risk for adverse health events and mortality independently of chronological age ^9^. The health variables included in a frailty index are routinely collected clinical data such as symptoms, signs, disabilities and diseases that meet standard criteria ^27^. As frailty index scores represent the proportion of total health deficits of an individual, higher scores indicate the accumulation of more age-related health deficits and worse health. For example, a person with 15 of 50 assessed health deficits has a frailty index score of 15/50 = 0.3.

Frailty index scores had been developed and validated previously in each cohort ^13,34–37^. Although these scores are generated from frailty indices composed of different health and functional deficits, frailty can be measured reliably if multiple physiological/functional domains are represented and if enough deficits (e.g. more than 30) are included ^27,38^. Fewer items can be included but the information reduces and measurement error increases accordingly ^39,40^. Where necessary, each frailty index was adapted for our investigation by ensuring that deficits closely reflecting cognition were removed from their composition, such as the diagnosis of a neurodegenerative disease or a measure of cognitive performance (Supplementary Table 1). For use in sensitivity analyses under objective 2 (i.e. when measuring the association of frailty and incident dementia), we calculated a second frailty index where we excluded deficits that were found to be independently associated with incident dementia based on analyses in each dataset.

Prior to using frailty index scores in survival models, the scores were multiplied by 10 so that hazard ratios could be meaningfully interpreted as the change in dementia risk associated with each 0.1 increase in frailty index scores.

### 2.4. Covariates

Consistent with previous work, participant age, sex and education level were included as covariates due to possibly confounding the relationship between frailty and incident dementia ^13^. In all datasets, age was measured in years at baseline; sex was a self-reported binary variable (male/female); education was reported at baseline and for consistency between studies was recoded into a three-category variable (lower, intermediate and higher education). In ELSA, higher education was completion of a higher education qualification below a degree, or a degree or equivalent. Intermediate education was completion of a CSE, GCE O, GCE A or equivalent, and lower education was no formal qualification. In HRS, higher education was completion of an associate’s degree, bachelor’s degree, master’s degree, PhD or similar. Intermediate education was completion of a high school diploma or GED, and lower education was no formal qualification. In MAP and NACC, higher education was more than 12 years of formal education, intermediate education was 10, 11 or 12 years of formal education, and lower education was less than 10 years of formal education. Information regarding mortality data, which were used in censoring, is included in Supplementary Information 2.

### 2.6. Statistical analysis

#### 2.6.1. Sample characteristics

The demographic characteristics of participants at baseline in each study were first summarised using descriptive statistics.

#### 2.6.2. Objective 1

To determine when an acceleration in the accumulation of frailty associated with impending dementia is first observable (objective 1), we modelled trajectories in frailty index scores (the dependent variable) using a backwards timescale. Here, a time value equalling zero was the year of incident dementia or censor and negative time values represented the number of years until that event. This approach has been used by others when exploring trajectories of dementia risk factors prior to dementia development ^21,22^.

For this process, we used the *Bayesian Regression Models using ‘Stan’* (*brms*) package in R to fit Bayesian generalised non-linear multilevel models ^41^. In each model, population-level effects of time were fitted using natural cubic splines, which allow for non-linear trajectories in frailty index scores (e.g. rate of increase in frailty may hasten with advancing age ^42^), and included both a random intercept and slope (linear fit) for participants. Preliminary models showed that six degrees of freedom (five knots) were appropriate parameters for the natural cubic spline of time; this aligns with recommendations that including more than six degrees of freedom in splines is often unnecessary even for large datasets (as analysed here) ^43^. Given the non-negative and right-skewed distribution of frailty index scores, we used the gamma distribution with a log link function.

A base model was first built that included fixed effects of time, event group (incident dementia or censored) and possible confounders (age, sex, education). We then built an interaction model to include an additional fixed effect representing the interaction between time (natural cubic spline) and event group (incident dementia or censored). This event group x time interaction term allowed the association of time and frailty index scores to vary by event group. Fit was compared between these two models to assess whether frailty trajectories differed between incident dementia and censored participants, with difference-in-fit statistics accompanied by 95% credible intervals to assist interpretation. From the interaction model, we assessed the marginal effect of event group on frailty trajectories by calculating expected frailty index scores for each participant at each time point while holding the other covariates constant (i.e. at each sample’s median age and the most frequently occurring level of each factor). These expected scores were plotted as trajectories stratified by dementia group. For greater specificity regarding the time point after which frailty accumulation consistently accelerated due to impending dementia, we calculated mean differences in expected scores by dementia group at each time point (rounded to nearest whole years) and tested these using t-tests. We estimated the start of the pre-dementia frailty acceleration period as the year after which the size of differences in frailty index scores between the incident dementia group and the censored group were observed to be statistically significant and increase consistently.

Convergence of four chains with each 3,000 iterations (excluding 500 warm-up iterations) under weakly informative priors was confirmed by inspection of trace plots and R-hat values. Standard model diagnostic tools (e.g. posterior predictive checks) were used to confirm the suitability of the modelling approach. Expected log pointwise predictive density (elpd) leave-one-out (loo) cross-validation was used to assess and compare model fit in all cases.

For objective 1, we defined the follow-up period as beginning at participants’ baseline assessments and continuing until incident dementia. In individuals who did not develop dementia, the follow-up ended three years before death or at the last date at which they were known to be without dementia, whichever came first. The three-year censoring rule was implemented to improve the comparison between frailty trajectories before incident dementia and frailty trajectories in normal ageing; this exclusion takes into account the known five-fold increase in the rate of health deficit accumulation that occurs within the last three years of life (often referred to as the “terminal decline” phase) ^34^. However, the three-year censoring rule could not be applied to ELSA due to unavailable mortality data (Supplementary Information 2).

#### 2.6.3. Objective 2

We next measured the association of frailty and incident dementia after controlling for any impact of the pre-dementia frailty acceleration period (objective 2). To do this, we first used Cox proportional hazards models to examine the relationships between frailty index scores and dementia risk while adjusting for possible confounders (age, sex and education) in the total samples. This model was then estimated separately within two subgroups. The first subgroup included participants whose time between baseline frailty measurement and event (incident dementia, censor) was less than or equal to the pre-dementia frailty acceleration period (as estimated in objective 1). The second subgroup included participants whose time between baseline frailty measurement and event was greater than the pre-dementia frailty acceleration period. Differences in the associations of frailty index scores with dementia risk between these groups were then quantified using interaction terms. Relationships were expressed as hazard ratios (HRs) and accompanied by 95% confidence intervals (CIs). For objective 2, the follow-up period additionally included the observations within three years of death for individuals who did not develop dementia.

#### 2.6.4. Sensitivity analyses

All statistical results were determined within the overall datasets and then within males and females, separately, within each dataset. These sex-stratified results are presented in Supplementary Figs. 1–4. For objective 2, two sensitivity analyses were conducted to assess the robustness of associations of frailty index scores and incident dementia. First, to ensure that the pre-dementia frailty acceleration period was not being systematically underestimated, it was increased by two years and analyses were repeated. Second, to reduce the potential that the inclusion of possibly confounding health deficits drove associations, analyses were repeated using a second frailty index that additionally excluded deficits shown to be independently associated (*P* < 0.05) with incident dementia in multivariable Cox proportional hazards models adjusted for age, sex, education and all other deficits.

#### 2.6.5. Analytical approach

We used a coordinated approach whereby the structure of datasets was first made consistent before an identical analytical procedure (Supplementary Analysis Script) was applied to generate summary statistics, statistical results and figures. All statistical analyses were conducted using R V.4.2.1.

## 3. RESULTS

### 3.1. Sample characteristics

Data from 23,672 participants (62% female) were included in this analysis (Table 1). Most participants were contributed by NACC (42%) and least by MAP (5%). In total, 242,760 person-years of follow-up and 2,906 cases of incident dementia were analysed. Among the cohorts, participants in MAP were oldest and had the highest degrees of frailty, on average, corresponding to the highest observed rates of incident dementia.

### 3.2. Frailty trajectories prior to dementia

**Figure 2:**
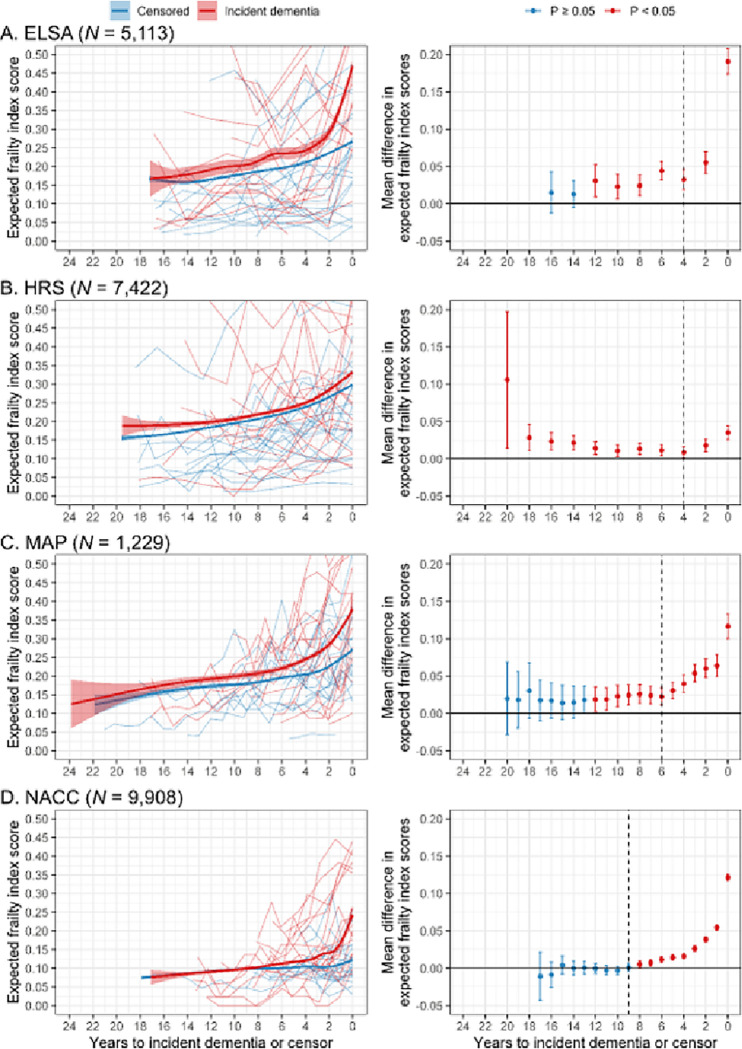
Frailty trajectories before dementia. Plots used expected frailty index scores calculated from Bayesian mixed-effects gamma regression models that included fixed effects of time, time x event group, age, sex and education, as well as random participant intercepts and slopes. For the trajectory plots, the thicker lines are mean trajectories surrounded by 95% credible intervals and the thinner lines represent raw (unadjusted) data from 20 participants randomly selected from each group. For the forest plots, mean differences (95% confidence intervals) are between the censored group (reference line) and the incident dementia group, and the dashed line represents the estimated start of the pre-dementia frailty acceleration period. ELSA, English Longitudinal Study of Ageing; HRS, Health and Retirement Study; MAP, Rush Memory and Aging Project; NACC, National Alzheimer’s Coordinating Center.

To determine when an acceleration in the accumulation of frailty associated with impending dementia might be first observable (objective 1), we modelled frailty index scores using backwards timescales and adjusted for potential confounders. In the years before incident dementia or censor, frailty index scores tended to increase (Fig. 2). Among the censored groups, gradual increases in frailty index scores were observed in all datasets, although these were smallest in NACC. Among the incident dementia groups, we observed accelerations in the rates of increase in frailty index scores in the years proximal to dementia. These were particularly pronounced in ELSA and NACC, and less so in MAP and HRS, although still present in those datasets. That divergence in frailty trajectories associated with incident dementia was supported by the model results, whereby, for all datasets, the inclusion of an event group (incident dementia or censored) by time interaction term resulted in improved model fit (Table 2). The population-level effects from the interaction model (i.e. that which included the event group by time interaction term) are presented in Supplementary Table 2.

Expected frailty index scores, calculated from the interaction model while holding the covariates of age, sex and education constant, were then compared between the incident dementia and censored groups at each year (Fig. 2). Compared with the censored groups, these frailty scores were consistently higher in the incident dementia groups, 20, 12, 12, and 8 years before dementia in HRS, ELSA, MAP and NACC, respectively. At the point of dementia detection, frailty index scores were most elevated in ELSA (0.19 points higher than censored participants), elevated to a similar degree in both MAP and NACC (0.12 points higher), and to a lesser extent in HRS (0.04 points higher). The start of the pre-dementia frailty acceleration period, i.e. the year after which the size of differences in frailty index scores between the incident dementia group and the censored group were observed to be statistically significant and increase consistently, was estimated at 9, 6, 4 and 4 years before dementia for NACC, MAP, ELSA and HRS, which was similar in both males and females (Supplementary Figs. 1–4). The mean differences in expected frailty index scores and associated *P* values are presented in Supplementary Table 3.

### 3.3. Frailty and incident dementia

We next measured the association of frailty index scores and incident dementia after controlling for the pre-dementia frailty acceleration period (objective 2). This we did by using Cox proportional-hazards models to determine the associations of frailty with incident dementia for participants whose time between baseline frailty measurement and event (incident dementia or censored) was greater than the cohort-specific pre-dementia frailty acceleration period (as estimated under objective 1). The size of analysed samples, the pre-dementia frailty acceleration periods, and the number of deficits included in frailty indices varied in the main and sensitivity analyses (Table 3).

**Figure 3:**
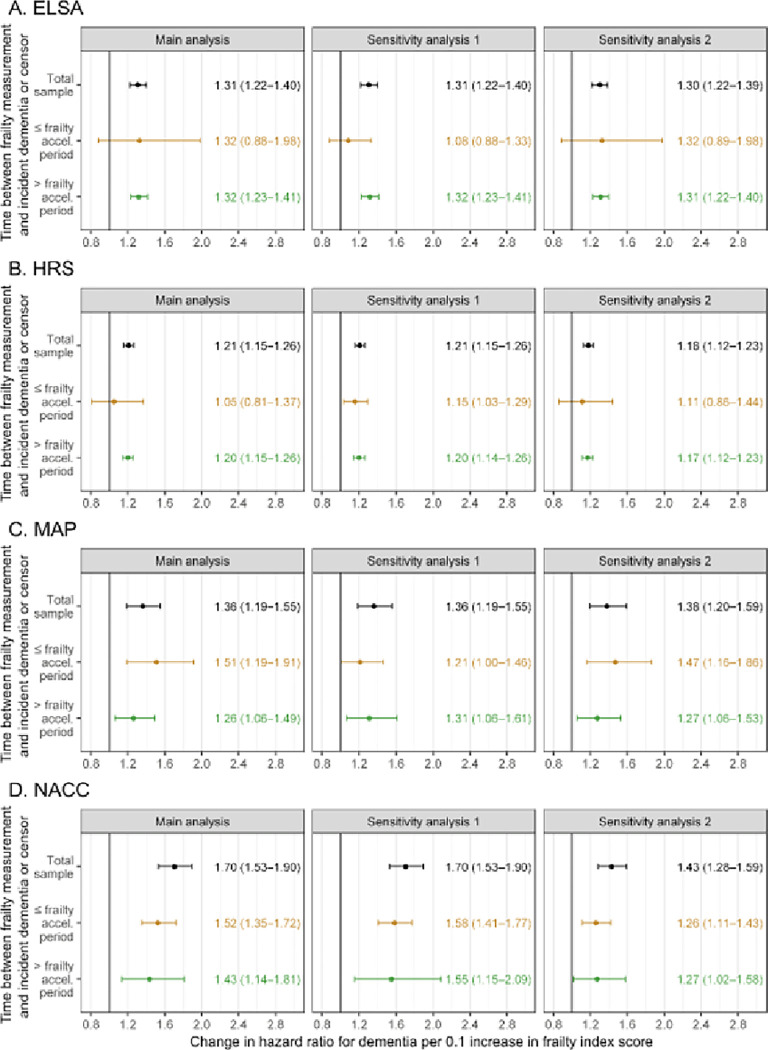
Associations of frailty and incident dementia. Hazard ratios were calculated from Cox proportional-hazards models that included covariates of age, sex and education. Sensitivity analysis 1, the pre-dementia frailty acceleration period was increased by two years; sensitivity analysis 2, deficits found to be independently associated with incident dementia were removed from the calculation of frailty index scores. Details regarding sizes of samples and subgroups included in these analyses are presented in Table 3. ELSA, English Longitudinal Study of Ageing, HRS, Health and Retirement Study; MAP, Rush Memory and Aging Project; NACC, National Alzheimer’s Coordinating Center.

In the main analyses, in each dataset, each 0.1 increase in frailty index scores (equivalent to 4–5 additional health deficits) was associated with higher dementia risk (Fig. 3). This association was strongest in NACC (70% increase in risk), weakest in HRS (21% increase in risk), and similar in ELSA (31% increase in risk) and MAP (36% increase in risk).

When the time between frailty measurement and incident dementia or censor was considered, associations remained similar in both groups (i.e. in participants whose time between frailty measurement and incident dementia or censor was less than or equal to the pre-dementia frailty acceleration period, and in participants whose time between measurement and outcome exceeded that period). Here, event timing x frailty index score interaction terms were not statistically significant in ELSA (*P* = 0.921), HRS (*P* = 0.205), MAP (*P* = 0.411) or NACC (*P* = 0.733). Across datasets and in participants whose baseline frailty measurement was conducted before the pre-dementia acceleration period had begun, the associations of frailty index scores with dementia risk were consistently positive and statistically significant. There, each 0.1 increase in frailty index scores was associated with 20–43% increased dementia risk, and in the absence of meaningful differences in this association between males and females (Supplementary Figs. 1–4). The results from both sensitivity analyses demonstrated a robustness in these findings, whereby frailty index scores calculated before the pre-dementia frailty acceleration period remained associated with incident dementia at a statistically significant level even when that period was extended by two years (sensitivity analysis 1). Likewise, our results were robust to removing health deficits that were independently associated with incident dementia from the calculation of frailty index scores (sensitivity analysis 2).

## 4. DISCUSSION

With the purpose of addressing reverse causality in the relationship between frailty and dementia, we identified the point at which frailty accelerated prior to dementia onset and determined how the timing of frailty measurement relative to that point affected the strength of risk associations. From this analysis of almost 24,000 individuals participating in four cohort studies in the United Kingdom and United States, we report three main findings: 1) an elevated degree of frailty was observed 8 to 20 years before dementia onset; 2) the rate of decline in health and function in prodromal dementia, as reflected in a higher degree of frailty, accelerated from 4–9 years before dementia onset; 3) frailty was a robust risk factor for incident dementia even when its measurement occurred before the pre-dementia frailty acceleration period. These results offer insight into the natural course of declining health in the subclinical stages of neurodegenerative diseases, position frailty index scores as a measure effective in identifying high-risk individuals for inclusion into treatment and prevention trials for dementia, and substantially strengthen the evidence for frailty serving as an upstream dementia risk factor.

Previous reports have suggested a preclinical phase of Alzheimer’s disease up to 15–20 years in length ^15,16^, with changes in health and function first detectable at a population level from 10 years before dementia onset. Examples of these include higher health care usage and lower social engagement (2 years prior to diagnosis) ^17,18^, accelerated cognitive decline (6–10 years prior) ^16,19^, and more depressive symptoms (10 years prior) ^20^. Instead of assuming a static inflection point for prodromal dementia in our attempts to investigate reverse causality, here we determined them dynamically within each dataset by modelling frailty trajectories. Even though we observed a degree of heterogeneity in frailty trajectories between the datasets, in each case the pre-dementia frailty acceleration period was estimated to lie within that 10-year prodromal period (ranging from 4–9 years), supporting those earlier studies. Consequently, one explanation for elevated frailty in the years proximal to dementia relates to the adverse impacts of neurodegenerative changes.

Aside from neurodegenerative processes hastening frailty accumulation, another explanation for our findings is that accelerated biological ageing is a dementia cause rather than consequence. In support, strong links have been established between changes in the hallmarks of ageing and the development of neurodegenerative diseases ^3,4^, and chronological age itself has long been understood as a key risk factor. Rapidly increasing frailty index scores, observed here up to 9 years before dementia onset, may therefore signal an exhaustion of systemic reserves leaving affected individuals vulnerable to diseases that might otherwise have remained subclinical ^9^. This loss of reserve associated with higher frailty has been demonstrated previously in dementia, where frailty was associated with weaker relationships between dementia and neuropathological burden and polygenic risk despite persistently high dementia rates ^8,44,45^.

Regardless of the nature of the relationship between the pre-dementia frailty acceleration period and subsequent dementia, the findings from our time-to-event analyses align with the position that frailty is a strong risk factor for dementia and that the relationship between frailty and dementia does not exclusively reflect reverse causality. In individuals whose measurement of frailty occurred before the pre-dementia frailty acceleration period had begun, and in both males and females, we observed each 0.1 increase in frailty index scores to increase dementia risk substantially. The strength of those associations with risk either remained the same (ELSA, HRS) or increased (MAP, NACC) in a sensitivity analysis that extended the pre-dementia frailty acceleration period by two years (sensitivity analysis 1), suggesting that frailty measurement conducted distally to the occurrence of dementia can be used for risk stratification. Those associations also remained statistically significant in a sensitivity analysis that calculated frailty index scores exclusively using deficits that were not independently associated with incident dementia (sensitivity analysis 2). Our findings join previous reports of a robust association between frailty and incident dementia, even when adjusting for a polygenic dementia risk score and a marker of area-level deprivation ^8^, adjusting for the competing risk of death ^12^, including only non-traditional risk factors in the composition of the frailty index ^14^, or when conceptualising frailty as a phenotype ^11^.

### 4.1. Strengths and limitations

A considerable strength of our investigation was the use of four different cohort studies across two continents, which varied in participant characteristics and in study methodologies. The setting of studies included retirement communities (MAP), national-level surveys (ELSA, HRS), and a multi clinic-based cohort (NACC), resulting in participant samples diverse in age, education level, degree of frailty, and rates of incident dementia. NACC participants were noteworthy in having the second highest rates of incident dementia despite the lowest degrees of frailty (relative to other cohorts), aligning with the known issues of NACC representativeness relative to the broader United States population (e.g. fewer physical and mental health problems but more subjective cognitive complaints) ^46^. The method of dementia detection employed in each study also varied substantially, from physician-derived diagnoses (MAP, NACC), to mostly self- and informant-report (ELSA), and to estimated classifications based on a combination of cognitive tests (HRS). Some studies used approximately annual interviews/assessments (MAP, NACC) while others were biennial (ELSA, HRS). These differences contributed to variability in our statistical findings, both in terms of the frailty trajectories and in the strength of associations between frailty index scores and incident dementia. Despite these differences, by applying a consistent analytical approach to each dataset and reviewing results independently, we observed an encouraging consistency in findings supportive of strong external validity.

Even so, our results should be interpreted with respect to a few limitations. 1) We applied a considered approach to reduce the possibility of reverse causality in the association of frailty and dementia, but it is unlikely that it can be ruled out entirely in the absence of a randomised design. Still, associations were observed consistently even when we overestimated the pre-dementia frailty acceleration period by two years. 2) For enhanced consistency and comparability in analyses between cohorts, we did not include potentially relevant covariates in statistical models unless they were universally available. Although we included education level, which is an important marker of socioeconomic status, we did not include other markers of social deprivation that may be causally associated with dementia ^47^. Similarly, genetic risk for dementia, often approximated using APOE ε4 status, was not adjusted for. Nonetheless, previous reports of strong associations between frailty and incident dementia even after adjusting for social deprivation (e.g. Townsend deprivation index) ^8^, and within both APOE ε4 carriers and non-carriers ^13^, lead us to maintain confidence in our findings. 3) The included cohort studies were from only two countries (United States and United Kingdom) and a characteristic of most cohort studies is a healthy participant selection bias. The extent to which our findings apply to non-Western populations, and to populations with fewer social resources and poorer health, is not yet known.

### 4.2. Conclusion

In conclusion, we found robust observational evidence that frailty increases dementia risk in a manner that appears independent of reverse causality. This study strengthens the evidence base for a causal association by producing novel evidence on the temporality of the relationship between frailty and incident dementia. These findings suggest that frailty measurements can be used to identify high-risk population groups for preferential enrolment into clinical trials for dementia prevention and treatment, and that frailty itself may represent a useful upstream target for behavioural and societal approaches to dementia prevention.

## Figures and Tables

**Table 1:** Characteristics of analytical samples

Characteristic	ELSA	HRS	MAP	NACC
*N*	5,113	7,422	1,229	9,908
Age at baseline, years				
Mean (*SD*)	68.9 (6.6)	69.1 (6.7)	78.5 (6.9)	72.7 (7.4)
Range	60–99	60–95	60–100	60–101
Sex, *N*(%)				
Male	2,268 (44)	2,928 (40)	286 (23)	3,444 (35)
Female	2,845 (56)	4,494 (61)	943 (77)	6,464 (65)
Education, *N*(%)				
Higher	1,168 (23)	1,761 (24)	925 (75)	8,324 (84)
Intermediate	1,763 (35)	4,199 (57)	267 (22)	1,386 (14)
Lower	2,182 (43)	1,462 (20)	37 (3)	198 (2)
Frailty index score at baseline				
Mean (SD)	0.14 (0.12)	0.16 (0.12)	0.18 (0.08)	0.09 (0.05)
Range	0.00–0.74	0.00–0.84	0.00–0.56	0.00–0.42
Number of repeat frailty measurements per participant				
Mean (SD)	4.8 (1.8)	6.1 (2.5)	7.2 (4.4)	5.6 (3.3)
Range	2–7	2–9	2–22	2–17
Incident dementia				
Number of cases (% absolute risk)	475 (9)	1,092 (15)	323 (26)	1,016 (10)
Dementia incidence rate per 100 person-years (person-years of follow-up)	0.8 (59,805)	1.1 (103,210)	3.1 (10,538)	1.5 (69,207)

Note: Proportions may not sum to 100% due to rounding. ELSA, English Longitudinal Study of Ageing; HRS, Health and Retirement Study; MAP, Rush Memory and Aging Project; NACC, National Alzheimer’s Coordinating Center. SD, standard deviation.

**Table 2:** Comparison of fit for Bayesian frailty trajectory models

Dataset	Expected log pointwise predictive density leave-one-out		
	Base model	Interaction model	Difference in fit
ELSA (*N* = 5,113)	42,839.2 (176.8)	42,972.9 (175.2)	133.7 (91.4, 176.0)
HRS (*N* = 7,422)	74,543.3 (239.0)	74,562.4 (238.8)	19.0 (1.36, 36.6)
MAP (*N* = 1,229)	15,306.2 (92.0)	15,354.4 (91.8)	48.2 (28.0, 68.4)
NACC (*N* = 9,906)	120,896.4 (247.2)	121,516.1 (244.9)	619.6 (542.8, 696.4)

Note: Higher values indicate better fit. For the base models and interaction models, values in brackets represent standard error. For the difference in fit, values in brackets represent 95% credible intervals. The base model included fixed effects of time (natural cubic spline), event group, age, sex and education. Model 2 included an additional interaction term between time x event group. Both models included random participant intercepts and slopes. ELSA, English Longitudinal Study of Ageing; HRS, Health and Retirement Study; MAP, Rush Memory and Aging Project; NACC, National Alzheimer’s Coordinating Center.

**Table 3:** Characteristics of frailty and incident dementia risk analyses

Dataset	Analysis	Pre-dementia frailty acceleration period, years before onset	Number of deficits included in frailty index	Number of participants analysed	Time between baseline frailty measurement and dementia or censor, number of participants (%)	
					≤ frailty acceleration period	> frailty acceleration period
ELSA	Main	4	51	5,113	81 (2)	5,032 (98)
	S1	6	51	5,113	294 (6)	4,819 (94)
	S2	4	47	5,113	81 (2)	5,032 (98)
HRS	Main	4	40	7,422	59 (1)	7,363 (99)
	S1	6	40	7,422	311 (5)	7,091 (96)
	S2	4	35	7,416	58 (1)	7,358 (99)
MAP	Main	6	41	1,229	426 (35)	803 (65)
	S1	8	41	1,229	643 (52)	586 (48)
	S2	6	33	1,107	390 (35)	717 (65)
NACC	Main	9	44	9,908	7,131 (72)	2,777 (28)
	S1	11	44	9,908	8,174 (83)	1,734 (18)
	S2	9	32	6,701	4,112 (61)	2,589 (39)

Note: The pre-dementia frailty acceleration period was estimated as the year after which the size of differences in frailty index scores between the incident dementia group and the censored group were observed to be statistically significant and increase consistently. S1, sensitivity analysis 1, in which the pre-dementia frailty acceleration period was increased by two years; S2, sensitivity analysis 2, whereby deficits found to be independently associated (*P* < 0.05) with incident dementia were removed from the calculation of frailty index scores. For S2, participants who did not have data on at least 30 items included in the second frailty index were excluded. ELSA, English Longitudinal Study of Ageing; HRS, Health and Retirement Study; MAP, Rush Memory and Aging Project; NACC, National Alzheimer’s Coordinating Center.

## References

[1] Boyle P. A. Attributable risk of Alzheimer’s dementia attributed to age-related neuropathologies. Ann Neurol 85, 114–124 (2019). 30421454 10.1002/ana.25380 PMC10128614

[2] Nichols E. The prevalence, correlation, and co-occurrence of neuropathology in old age: harmonisation of 12 measures across six community-based autopsy studies of dementia. Lancet Healthy Longev 4, e115–e125 (2023). 36870337 10.1016/S2666-7568(23)00019-3 PMC9977689

[3] Hou Y. Ageing as a risk factor for neurodegenerative disease. Nat Rev Neurol 15, 565–581 (2019). 31501588 10.1038/s41582-019-0244-7

[4] Melo Dos Santos L. S., Trombetta-Lima M., Eggen B. & Demaria M. Cellular senescence in brain aging and neurodegeneration. Ageing Res Rev 93, 102141 (2023). 38030088 10.1016/j.arr.2023.102141

[5] Gonzales M. M. Senolytic therapy in mild Alzheimer’s disease: a phase 1 feasibility trial. Nat Med 29, 2481–2488 (2023). 37679434 10.1038/s41591-023-02543-w PMC10875739

[6] Gonçalves R. S. D. S. A., Maciel Á. C. C., Rolland Y., Vellas B. & de Souto Barreto P. Frailty biomarkers under the perspective of geroscience: A narrative review. Ageing Res Rev 81, 101737 (2022). 36162706 10.1016/j.arr.2022.101737

[7] Diebel L. W. M. & Rockwood K. Determination of Biological Age: Geriatric Assessment vs Biological Biomarkers. Curr Oncol Rep 23, 104 (2021). 34269912 10.1007/s11912-021-01097-9 PMC8284182

[8] Ward D. D., Ranson J. M., Wallace L. M. K., Llewellyn D. J. & Rockwood K. Frailty, lifestyle, genetics and dementia risk. J Neurol Neurosurg Psychiatry 93, 343–350 (2022). 34933996 10.1136/jnnp-2021-327396 PMC8921595

[9] Howlett S. E., Rutenberg A. D. & Rockwood K. The degree of frailty as a translational measure of health in aging. Nat Aging 1, 651–665 (2021). 37117769 10.1038/s43587-021-00099-3

[10] Blodgett J. M. Prognostic accuracy of 70 individual frailty biomarkers in predicting mortality in the Canadian Longitudinal Study on Aging. Geroscience 46, 3061–3069 (2024). 38182858 10.1007/s11357-023-01055-2 PMC11009196

[11] Buchman A. S., Boyle P. A., Wilson R. S., Tang Y. & Bennett D. A. Frailty is associated with incident Alzheimer’s disease and cognitive decline in the elderly. Psychosom Med 69, 483–489 (2007). 17556640 10.1097/psy.0b013e318068de1d

[12] Rogers N. T., Steptoe A. & Cadar D. Frailty is an independent predictor of incident dementia: Evidence from the English Longitudinal Study of Ageing. Sci Rep 7, 15746 (2017). 29146957 10.1038/s41598-017-16104-y PMC5691042

[13] Ward D. D., Wallace L. M. K. & Rockwood K. Cumulative health deficits, APOE genotype, and risk for later-life mild cognitive impairment and dementia. J Neurol Neurosurg Psychiatry 92, 136–142 (2021). 33188132 10.1136/jnnp-2020-324081 PMC7841490

[14] Song X., Mitnitski A. & Rockwood K. Nontraditional risk factors combine to predict Alzheimer disease and dementia. Neurology 77, 227–234 (2011). 21753161 10.1212/WNL.0b013e318225c6bc PMC3136058

[15] Dubois B. Preclinical Alzheimer’s disease: Definition, natural history, and diagnostic criteria. Alzheimers Dement 12, 292–323 (2016). 27012484 10.1016/j.jalz.2016.02.002 PMC6417794

[16] Jia J. Biomarker Changes during 20 Years Preceding Alzheimer’s Disease. N Engl J Med 390, 712–722 (2024). 38381674 10.1056/NEJMoa2310168

[17] Zhu C. W. Medicare Utilization and Expenditures Around Incident Dementia in a Multiethnic Cohort. J Gerontol A Biol Sci Med Sci 70, 1448–1453 (2015). 26311543 10.1093/gerona/glv124 PMC4612389

[18] Hackett R. A., Steptoe A., Cadar D. & Fancourt D. Social engagement before and after dementia diagnosis in the English Longitudinal Study of Ageing. PLoS One 14, e0220195 (2019). 31369590 10.1371/journal.pone.0220195 PMC6675105

[19] Li G. Cognitive Trajectory Changes Over 20 Years Before Dementia Diagnosis: A Large Cohort Study. J Am Geriatr Soc 65, 2627–2633 (2017). 28940184 10.1111/jgs.15077 PMC5729097

[20] Singh-Manoux A. Trajectories of Depressive Symptoms Before Diagnosis of Dementia: A 28-Year Follow-up Study. JAMA Psychiatry 74, 712–718 (2017). 28514478 10.1001/jamapsychiatry.2017.0660 PMC5710246

[21] Singh-Manoux A. Obesity trajectories and risk of dementia: 28 years of follow-up in the Whitehall II Study. Alzheimers Dement 14, 178–186 (2018). 28943197 10.1016/j.jalz.2017.06.2637 PMC5805839

[22] Sabia S. Physical activity, cognitive decline, and risk of dementia: 28 year follow-up of Whitehall II cohort study. BMJ 357, j2709 (2017). 28642251 10.1136/bmj.j2709 PMC5480222

[23] Steptoe A., Breeze E., Banks J. & Nazroo J. Cohort profile: the English longitudinal study of ageing. Int J Epidemiol 42, 1640–1648 (2013). 23143611 10.1093/ije/dys168 PMC3900867

[24] Sonnega A. Cohort Profile: the Health and Retirement Study (HRS). Int J Epidemiol 43, 576–585 (2014). 24671021 10.1093/ije/dyu067 PMC3997380

[25] Bennett D. A. Religious Orders Study and Rush Memory and Aging Project. J Alzheimers Dis 64, S161–S189 (2018). 29865057 10.3233/JAD-179939 PMC6380522

[26] Beekly D. L. The National Alzheimer’s Coordinating Center (NACC) database: the Uniform Data Set. Alzheimer Dis Assoc Disord 21, 249–258 (2007). 17804958 10.1097/WAD.0b013e318142774e

[27] Theou O., Haviva C., Wallace L., Searle S. D. & Rockwood K. How to construct a frailty index from an existing dataset in 10 steps. Age and Ageing 52, afad221 (2023). 38124255 10.1093/ageing/afad221 PMC10733590

[28] Wingo T. S., Lah J. J., Levey A. I. & Cutler D. J. Autosomal recessive causes likely in early-onset alzheimer disease. Arch Neurol 69, 59–64 (2012). 21911656 10.1001/archneurol.2011.221 PMC3332307

[29] Burton J. K. Informant Questionnaire on Cognitive Decline in the Elderly (IQCODE) for the detection of dementia within a secondary care setting. Cochrane Database Syst Rev 7, CD010772 (2021). 34278561 10.1002/14651858.CD010772.pub3 PMC8406705

[30] Langa K. M., Weir D. R., Kabeto M. & Sonnega A. Langa-Weir Classification of Cognitive Function (1995 Onward). (2020).

[31] McKhann G. Clinical diagnosis of Alzheimer’s disease: report of the NINCDS-ADRDA Work Group under the auspices of Department of Health and Human Services Task Force on Alzheimer’s Disease. Neurology 34, 939–944 (1984). 6610841 10.1212/wnl.34.7.939

[32] American Psychiatric Association. Diagnostic and Statistical Manual of Mental Disorders: DSM-IV. (American Psychiatric Association, Washington, DC, 1994).

[33] Dent E., Chapman I., Howell S., Piantadosi C. & Visvanathan R. Frailty and functional decline indices predict poor outcomes in hospitalised older people. Age Ageing 43, 477–484 (2014). 24257468 10.1093/ageing/aft181

[34] Stolz E. Acceleration of health deficit accumulation in late-life: evidence of terminal decline in frailty index three years before death in the US Health and Retirement Study. Ann Epidemiol 58, 156–161 (2021). 33812966 10.1016/j.annepidem.2021.03.008

[35] Pugh C., Eke C., Seth S., Guthrie B. & Marshall A. Frailty before and during austerity: A time series analysis of the English Longitudinal Study of Ageing 2002–2018. PLoS One 19, e0296014 (2024). 38324538 10.1371/journal.pone.0296014 PMC10849239

[36] Wallace L. M. K. 10-year frailty trajectory is associated with Alzheimer’s dementia after considering neuropathological burden. Aging Med (Milton) 4, 250–256 (2021). 34964005 10.1002/agm2.12187 PMC8711220

[37] Yang Y. & Lee L. C. Dynamics and heterogeneity in the process of human frailty and aging: evidence from the U.S. older adult population. J Gerontol B Psychol Sci Soc Sci 65B, 246–255 (2010). 20007299 10.1093/geronb/gbp102 PMC2981448

[38] Stolz E. Reliability of the frailty index among community-dwelling older adults. The Journals of Gerontology: Series A glad 227 (2023) doi:10.1093/gerona/glad227. PMC10809054 37738215

[39] Farrell S. G., Mitnitski A. B., Rockwood K. & Rutenberg A. D. Network model of human aging: Frailty limits and information measures. Phys. Rev. E 94, 052409 (2016). 27967091 10.1103/PhysRevE.94.052409

[40] Nguyen Q. D., Moodie E. M., Keezer M. R. & Wolfson C. Clinical Correlates and Implications of the Reliability of the Frailty Index in the Canadian Longitudinal Study on Aging. The Journals of Gerontology: Series A 76, e340–e346 (2021). 10.1093/gerona/glab161 PMC8514068 34097017

[41] Bürkner P.-C. brms: An R Package for Bayesian Multilevel Models Using Stan. Journal of Statistical Software 80, 1–28 (2017).

[42] Kulminski A. Accelerated accumulation of health deficits as a characteristic of aging. Experimental Gerontology 42, 963–970 (2007). 17601693 10.1016/j.exger.2007.05.009 PMC2096614

[43] Harrell F. E. Jr. Regression Modeling Strategies: With Applications to Linear Models, Logistic and Ordinal Regression, and Survival Analysis. (Springer International Publishing, 2015). doi:10.1007/978-3-319-19425-7.

[44] Wallace L. M. K. Investigation of frailty as a moderator of the relationship between neuropathology and dementia in Alzheimer’s disease: a cross-sectional analysis of data from the Rush Memory and Aging Project. Lancet Neurol 18, 177–184 (2019). 30663607 10.1016/S1474-4422(18)30371-5 PMC11062500

[45] Canevelli M. Biomarkers and phenotypic expression in Alzheimer’s disease: exploring the contribution of frailty in the Alzheimer’s Disease Neuroimaging Initiative. Geroscience 43, 1039–1051 (2021). 33210215 10.1007/s11357-020-00293-y PMC8110661

[46] Arce Rentería M. Representativeness of samples enrolled in Alzheimer’s disease research centers. Alzheimers Dement (Amst) 15, e12450 (2023). 37287650 10.1002/dad2.12450 PMC10242202

[47] Daly T. Dementia Prevention Guidelines Should Explicitly Mention Deprivation. AJOB Neuroscience 15, 73–76 (2024). 37379079 10.1080/21507740.2023.2225461

